# A 2‐Year Randomized Controlled Trial With Low‐Dose B‐Vitamin Supplementation Shows Benefits on Bone Mineral Density in Adults With Lower B12 Status

**DOI:** 10.1002/jbmr.4709

**Published:** 2022-10-14

**Authors:** Michelle Clements, Maria Heffernan, Mary Ward, Leane Hoey, Leanne C Doherty, Roberta Hack Mendes, Michelle M Clarke, Catherine F Hughes, Ingrid Love, Shauna Murphy, Eilish McDermott, Jennifer Grehan, Adrian McCann, Liadhan B McAnena, JJ Strain, Lorraine Brennan, Helene McNulty

**Affiliations:** ^1^ Nutrition Innovation Centre for Food and Health (NICHE), School of Biomedical Sciences Ulster University Coleraine Northern Ireland UK; ^2^ UCD School of Agriculture and Food Science Institute of Food and Health, Conway Institute, University College Dublin Dublin Ireland; ^3^ Section of Radiography and Diagnostic Imaging, School of Medicine University College Dublin Dublin Ireland; ^4^ BEVITAL AS Bergen Norway

**Keywords:** B‐VITAMINS, ONE‐CARBON METABOLISM, BONE MINERAL DENSITY, OSTEOPOROSIS, AGING

## Abstract

Folate, vitamins B12, B6, and riboflavin are required for one‐carbon metabolism and may affect bone health, but no previous randomized trial has investigated all four nutrients in this context. We investigated the effect of low‐dose B‐vitamins for 2 years on bone mineral density (BMD) in a dual‐centered, 2‐year randomized controlled trial (RCT) in adults aged ≥50 years. Eligible participants not consuming B‐vitamin supplements or fortified foods >4 times weekly were randomized to receive daily either combined folic acid (200 μg), vitamin B12 (10 μg), vitamin B6 (10 mg), and riboflavin (5 mg), or “active” placebo, whereby both the intervention and placebo groups received vitamin D (10 μg). BMD was assessed before and after intervention using dual‐energy X‐ray absorptiometry (DXA) scanning of the total hip, femoral neck, and lumbar spine (L1 to L4). Of 205 eligible participants randomized, 167 completed the trial in full. B‐vitamin intervention resulted in increases in serum folate (*p* < 0.001), serum B12 (*p* < 0.001), and plasma pyridoxal‐5‐phosphate (*p* < 0.001) and decreases in functional biomarkers of B‐vitamin status, erythrocyte glutathione reductase activation coefficient (*p* < 0.001), serum methylmalonic acid (MMA; *p* < 0.001), and serum total homocysteine (*p* < 0.001). B‐vitamin intervention had no overall effect on BMD, which declined in both treatment groups by approximately 1% (ranging from −0.7% to −1.4%). However, in participants with lower baseline B12 status (serum B12 <246 pmol/L or MMA ≥0.22 μmol/L), B‐vitamin intervention reduced the 2‐year BMD decline versus placebo: adjusted mean (95% confidence interval [CI]) change of −0.003 (−0.008, 0.002) versus −0.015 (−0.021, −0.010) g/cm^2^ at the total hip and −0.004 (−0.010, 0.001) versus −0.013 (−0.018, −0.007) g/cm^2^ at the femoral neck. In conclusion, the findings indicate that although low‐dose B‐vitamin intervention for 2 years had no overall effect on BMD, improving B‐vitamin status appears to have specific benefits for bone health in adults with lower B12 status. © 2022 The Authors. *Journal of Bone and Mineral Research* published by Wiley Periodicals LLC on behalf of American Society for Bone and Mineral Research (ASBMR).

## Introduction

Osteoporosis, characterized by a reduction in bone mineral density (BMD) and deterioration of bone microarchitecture,^(^ ^1^ ^)^ represents a substantial public health challenge that affects more than 200 million people globally.^(^ ^2^ ^)^ Osteoporotic fractures are a particular source of disability in older adults^(^ ^3^ ^)^ and carry a huge economic burden, with costs estimated at more than €56 billion annually in Europe alone.^(^ ^4^ ^)^ Nutrition is a key modifiable factor in the development of osteoporosis and an obvious cost‐effective measure for maintaining adequate bone health and preventing the onset of osteoporosis. Although calcium and vitamin D have well‐established roles in maintaining BMD^(^ ^5^, ^6^ ^)^ and preventing osteoporotic fracture,^(^ ^7^ ^)^ there is international evidence to suggest protective roles of folate, vitamin B12, and the interrelated B‐vitamins, as well as potential adverse effects of the related metabolite, homocysteine, in bone health.

Folate and vitamin B12 are essential in one‐carbon metabolism and are the main determinants of homocysteine; a low status of either vitamin leads to elevated plasma homocysteine.^(^ ^8^, ^9^ ^)^ Higher homocysteine concentrations, in turn, have been linked with lower BMD and higher fracture risk in older adults.^(^ ^10^ ^)^ Other evidence suggests an independent role for vitamin B12 in bone health. Notably, the Framingham Osteoporosis Study reported that deficient plasma B12 concentrations (<148 pmol/L) were associated with lower BMD.^(^ ^11^ ^)^ Likewise, among celiac disease patients in Northern Ireland, serum vitamin B12 was found to be a significant determinant of hip BMD in males.^(^ ^12^ ^)^ Furthermore, vitamin B12 deficiency commonly occurs in older people, primarily owing to food‐bound B12 malabsorption related to atrophic gastritis and widespread use of proton‐pump inhibitor medications (PPIs), rather than as a result of low dietary B12 intakes in higher‐income countries.^(^ ^8^, ^13^ ^)^ We recently reported high rates of vitamin B12 deficiency in older Irish adults, most notably among those with atrophic gastritis or, to a lesser extent, taking PPIs.^(^ ^14^ ^)^ With regard to folate, analysis of the Hordaland Homocysteine Study showed that lower plasma folate was associated with low BMD^(^ ^15^ ^)^ and increased fracture risk^(^ ^16^ ^)^ among Norwegian women. Although much less well studied compared with folate and B12, vitamin B6 and riboflavin are also required for one‐carbon metabolism, with some evidence of their potential role in bone health. Vitamin B6 deficiency has also been associated with poor bone health, with reports of greater mean bone loss in B6‐deficient older American adults,^(^ ^17^ ^)^ whereas higher dietary B6 intakes were linked with reduced osteoporotic fracture risk.^(^ ^18^ ^)^ In addition, large cohort studies have shown that low dietary riboflavin is associated with lower BMD and higher fracture risk when it occurs in combination with the variant 677TT genotype in the gene encoding methylenetetrahydrofolate reductase (MTHFR), a key folate‐metabolizing enzyme that requires riboflavin (FAD) as a co‐factor for its activity.^(^ ^19^, ^20^ ^)^

Despite the observational evidence linking low status of one or more of these interrelated B‐vitamins with an increased risk of osteoporotic fracture^(^ ^16^ ^)^ and/or lower BMD,^(^ ^11^, ^15^ ^)^ there are few randomized trials examining these associations, and no previous study has investigated the effect of all four aforementioned B‐vitamins. The aim of this trial was to investigate the effect of combined low‐dose B‐vitamin supplementation for 2 years on BMD in community‐dwelling adults. We hypothesized that intervention to optimize B‐vitamin status would benefit bone, particularly in those with lower baseline status of vitamin B12 and/or related B‐vitamins.

## Subjects and Methods

### Study participants

The Optimal Nutrition for Healthier Aging (OptiAge) study was a dual‐centered randomized trial based in Ulster University (UU), Coleraine, Northern Ireland, and University College Dublin (UCD), Republic of Ireland. Ethical approval was granted at each center by the relevant ethics committees (UU: Office for Research Ethics Committees Northern Ireland, [ORECNI], reference 08/NIR03/113; UCD: LS‐16‐62‐Clarke‐Brennan, ClinicalTrials.gov identifier NCT03892395) and all participants provided written informed consent at the time of recruitment. Participants at both centers were initially sampled between 2017 and 2019. At UU, the OptiAge trial was conducted in participants who had previously been recruited to the Trinity‐Ulster‐Department of Agriculture (TUDA) cohort study and were available for resampling. As described in detail elsewhere,^(^ ^21^ ^)^ TUDA involved community‐dwelling older adults (*n* = 5186) recruited to the study between 2008 and 2012 from Northern Ireland and the Republic of Ireland. At UCD, OptiAge participants were recruited de novo through posters displayed around the university campus and surrounding areas, notices in local church newsletters, and contacting community groups. Interested individuals were provided with the participant information sheet and allowed time to consider taking part.

Potential participants at both centers were screened for eligibility using a short questionnaire to exclude those likely to have high B‐vitamin biomarkers owing to dietary supplements and fortified foods. Participants were included if they were aged ≥50 years and reported that they were not currently taking a supplement containing folic acid or related B‐vitamins, or consuming >4 portions of B‐vitamin‐fortified food per week consumption levels previously shown to contribute to high B‐vitamin concentrations.^(^ ^21^ ^)^ Participants were also excluded if they were receiving vitamin B12 injections, had a diagnosis of celiac disease, Crohn's disease, ulcerative colitis, non‐alcoholic fatty liver disease, hepatitis or chronic obstructive pulmonary disease, or were taking any medications known to interfere with folate and/or B‐vitamin metabolism.

**Fig. 1:**
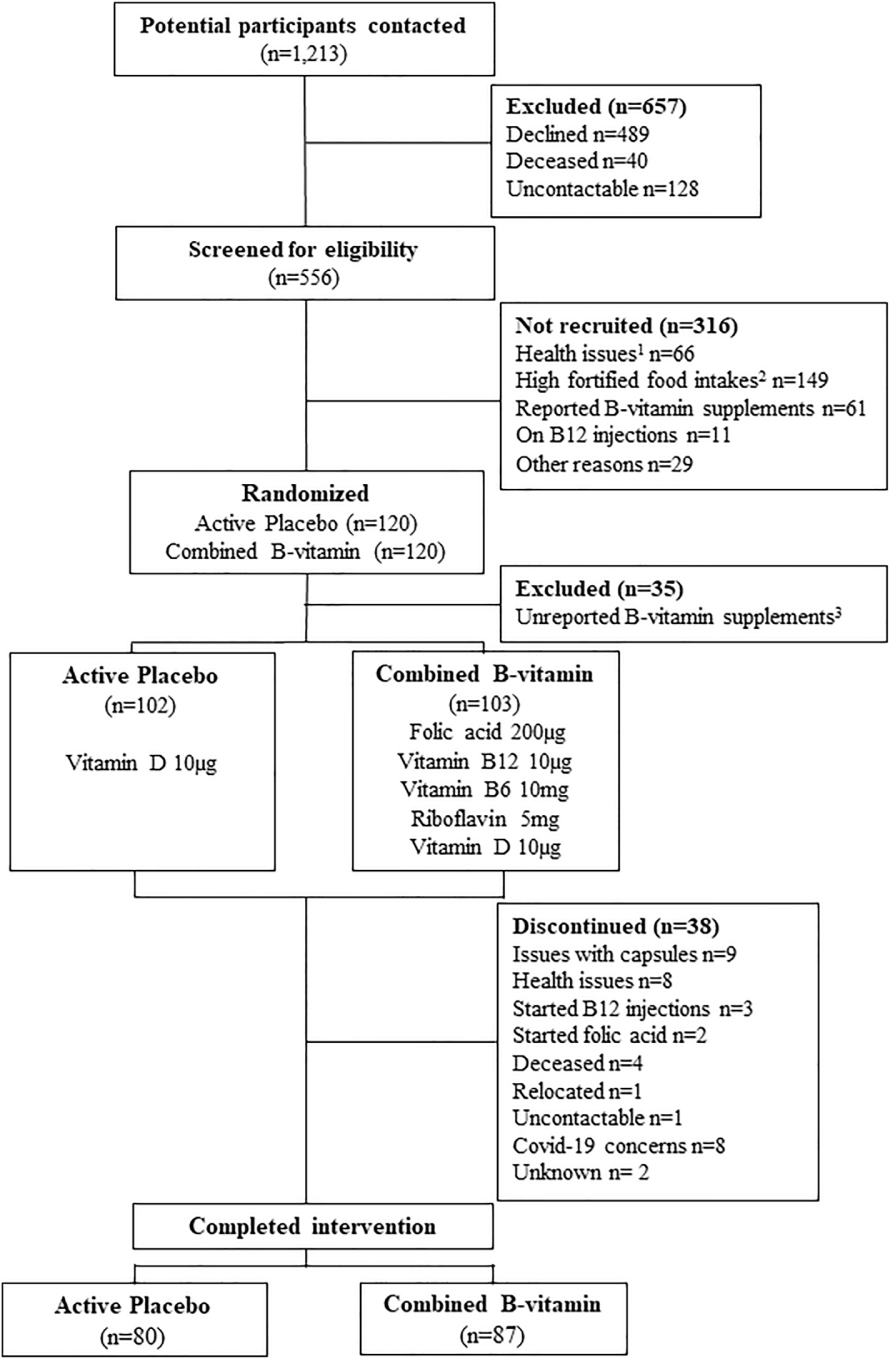
CONSORT flow diagram of participants from the OptiAge trial. ^1^Health issues include preexisting diagnosis of celiac disease, Crohn's disease, ulcerative colitis, liver disease, or chronic obstructive pulmonary disease. ^2^Participant consumed ≥4 portions (per week) of food fortified with B‐vitamins. ^3^Identified as unreported B‐vitamin supplement user after analysis of B‐vitamin biomarkers.

Our a priori study protocol included an additional step that was implemented after completion of the trial to retrospectively confirm participant eligibility, given that self‐reported dietary supplement usage is prone to error. Rather than relying on self‐reported dietary supplement usage only, we applied biomarker evidence at the time of analysis to identify participants who had been taking a B‐vitamin supplement on the basis of having a biomarker value in the range that could not be achieved through dietary sources only.^(^ ^21^ ^)^ Using this approach, we identified a small number of additional participants considered to have underreported supplement use at the time of screening and sampling, who were retrospectively deemed ineligible for participation in this trial (Fig. 1).

### Study design and compliance

This study was conducted as a double‐blinded, placebo‐controlled RCT (Fig. 1). Eligible participants were stratified by recruitment center, sex, and the lowest *T*‐score at any measured bone site, and subsequently randomized from within each stratum to receive combined folic acid (200 μg), vitamin B12 (10 μg), vitamin B6 (10 mg), and riboflavin (5 mg) or “active” placebo, whereby both the intervention and placebo groups received vitamin D (10 μg) for the 2‐year intervention period. Given the known beneficial effects of vitamin D supplementation on bone health, both treatment groups received vitamin D, therefore ensuring that any observed benefits of intervention on BMD could be attributed to B‐vitamin intervention. The B‐vitamin doses selected for this trial, although within the dietary ranges, were somewhat higher (with the exception of folate) than both recommended and habitual intakes of these B‐vitamins in Irish and Northern Irish adults,^(^ ^22^ ^)^ but nonetheless within the range achievable via a combination of natural and fortified food sources. All aspects of randomization were carried out by the Human Interventions Studies Unit (HISU) Clinical Trials Manager at UU.

The study supplements were provided by Advanced Orthomolecular Research (AOR; Calgary, Canada) and their composition at baseline was independently analyzed by ALS Life Sciences Ireland (Tipperary, Ireland) and Bio‐Chem Consulting Services Ltd (Calgary, Canada). The treatment and placebo capsules were administered daily and were identical in appearance and taste and were packaged in identical blister packs. To maximize compliance, capsules were provided in eight batches over the 2 years, each providing a 3‐month supply. Participants were asked to return all blister packs, including any untaken capsules, to estimate compliance to the intervention by pill counting. Participants were invited to attend two 90‐minute appointments at the beginning and end of the trial at either the HISU at UU, Coleraine; the Clinical Translational Research and Innovation Centre at Altnagelvin Area Hospital, Londonderry; Melrose Clinic, Londonderry; or UCD, Dublin. Owing to the COVID‐19 pandemic, ethical approval was sought at both centers to extend the original 2‐year intervention period by up to 6 months until COVID‐19 restrictions became relaxed, thus enabling participants to attend post‐intervention study visits and bone scans.

### BMD measurement

BMD was measured at the total hip, femoral neck, and lumbar spine (L1 to L4) by DXA (Lunar iDXA, GE Healthcare, Bucks, UK), using standardized protocols. A fully trained operator performed DXA scans, and a single radiographer evaluated all the scans. Results were expressed as grams of BMD per square centimeter (g/cm^2^), and as *T*‐scores. Using the World Health Organization criteria,^(^ ^23^ ^)^ participants were classified as normal, osteopenic, or osteoporotic based on *T*‐scores of ≥−1.0 standard deviation (SD), between −1.0 SD and −2.5 SD, and ≤−2.5 SD, respectively, and overall classification was based on the lowest *T*‐score at any of the measured sites. The densitometer between sites showed high precision with a percentage coefficient of variation (CV) for phantom spine scans of <1%.

### General health, lifestyle, and biophysical measures

Health and lifestyle information was obtained using a researcher‐assisted questionnaire, which included information on smoking, alcohol intake, medical history and medication use, supplement use, and physical activity levels, determined by the Physical Activity Scale for the Elderly.^(^ ^24^ ^)^ Anthropometric measurements, including weight, height, waist, and hip circumference, were recorded and blood pressure measurements were taken in accordance with standard operating procedures by a fully trained researcher. The Timed Up and Go test was measured to assess functional mobility.^(^ ^25^ ^)^ The Physical Self‐Maintenance Scale, and the Instrumental Activities of Daily Living scale were used to assess the general ability of participants to function independently.

### Dietary assessment

Dietary intakes were evaluated using a 4‐day food diary (over 4 consecutive days, including Saturday and Sunday, to account for the known variation in day‐to‐day intake) in combination with a researcher‐assisted food frequency questionnaire (FFQ), a method previously validated for assessing B‐vitamin intake from natural and fortified food sources using B‐vitamin biomarkers.^(^ ^26^ ^)^ Nutrient intakes were analyzed using the Nutritics nutritional software package (v. 4), which was customized to include the most recent nutritional content of B‐vitamin‐fortified food products. Mean daily energy and B‐vitamin intakes were calculated for each participant.

### Blood sampling and laboratory analysis

At both time points (pre‐ and post‐intervention), a non‐fasting (50 mL) blood sample was obtained from each participant on the day of sampling. For research biomarkers, all sample preparation and fractionation were carried out within 4 hours of collection, and blood aliquots were stored at −70°C until batch analysis at the end of the trial. B‐vitamin biomarkers were analyzed centrally using established methods at BEVITAL AS (Bergen, Norway; vitamin B12, folate, total homocysteine [tHcy], methylmalonic acid [MMA]) or UU, Coleraine (vitamin B6, riboflavin). Microbiological assays based on a chloramphenicol‐resistant strain of *Lactobacillus casei* ^(^ ^27^ ^)^ and colistin sulphate‐resistant strain of *Lactobacillus leichmannii* ^(^ ^28^ ^)^ were used to determine serum folate and serum total vitamin B12 concentrations, respectively. Serum concentrations of tHcy and MMA were analyzed by gas chromatography‐tandem mass spectrometry based on methylchloroformate derivatization.^(^ ^29^ ^)^ A combined indicator of vitamin B12 (cB12) status was calculated using serum total vitamin B12, MMA, and tHcy concentrations, with the value corrected for age and serum folate concentrations as described elsewhere.^(^ ^30^, ^31^ ^)^ A cB12 indicator value of ≤−0.5 was defined as low and deficient status. Plasma pyridoxal‐5‐phosphate (PLP), a marker of vitamin B6 status, was analyzed using high‐performance liquid chromatography with fluorescence detection.^(^ ^32^ ^)^ Riboflavin status (vitamin B2) was measured by erythrocyte glutathione reductase activation coefficient (EGRac), which is widely accepted as the gold‐standard measure of riboflavin status.^(^ ^33^ ^)^ This functional assay measures the activity of glutathione reductase before and after in vitro reactivation with its prosthetic group flavin adenine dinucleotide; results are reported as a ratio, whereby increasing EGRac ratio is indicative of decreasing riboflavin status. Vitamin D was analyzed as 25‐hydroxyvitamin D (25(OH)D) in the biochemistry laboratory at St James's Hospital, Dublin. Serum 25(OH)D was measured by liquid chromatography mass spectrometry.^(^ ^34^ ^)^

For each assay, quality controls were provided by the repeated analysis of pooled samples, and all samples were analyzed blind. The within‐ and between‐day CV was 4% and 5%, for both serum folate and serum B12. For tHcy and MMA, the within‐day CV ranged from 1% to 2% and the between‐day CV ranged from 2% to 3%. For PLP, the within‐day CV was 6% for QC1 and 5% for QC2, and the between‐day CV was 8% for QC1 and 4% for QC2. For EGRac, both the within‐ and between‐day CVs for the low and high riboflavin QCs was 2%.

### Sample size justification

Of the four B‐vitamins used in this trial, vitamin B12 was selected to estimate the required sample size because B12 has been most widely investigated in relation to bone health. The sample size required for this trial was calculated, before recruitment and randomization, using published data reporting vitamin B12 concentrations in relation to BMD in the Framingham Offspring Osteoporosis Study cohort.^(^ ^11^ ^)^ The sample size was estimated from the difference (by 0.026 g/cm^2^) in mean BMD values observed at the total hip in female participants with plasma vitamin B12 concentration in the highest compared with the lowest category (ie, >259 pmol/L versus ≤148 pmol/L). A sample size of 76 subjects per treatment group was estimated with a power of 80% and at a significance level (α) of 0.05. To allow for a high dropout rate, which was anticipated over the 2‐year intervention period and given the older age of study participants, the target sample size was increased, leading to a total of 240 participants recruited (ie, 120 participants per treatment group) and allowing for 40% dropout.

### Statistical analysis

All statistical analyses were performed using SPSS software, version 27.0 (SPSS UK Ltd, Chersey, UK). Statistical analyses were performed blind without the researchers knowing the treatment allocation of study participants. Data were analyzed on an intention‐to‐treat basis, and any missing data at post‐intervention were imputed before analysis, using the approach of carrying forward the baseline value for any missing value. Data were checked for normality and log‐transformed where appropriate. Differences in baseline characteristics between treatment groups were compared with chi‐square tests for categorical data and independent *t* tests or analysis of covariance (ANCOVA; adjusted for age) for continuous data. The determinants of baseline BMD at each measured site (total hip, femoral neck, lumbar spine) were examined using multiple linear regression. The risk of osteopenia/osteoporosis was evaluated using binary logistic regression.

B‐vitamin biomarker responses to intervention were investigated by repeated measures analysis of variance (ANOVA), with “time” (within‐subject factor: pre‐ versus post‐intervention) and “treatment” (between subject factor; treatment versus “active” placebo group) interaction. To assess the overall effect of B‐vitamin intervention on BMD, ANCOVA (adjusted for age, sex, body mass index [BMI], alcohol) was conducted. Subgroup analyses were performed using ANCOVA (adjusted for age and sex) to compare the BMD decline between groups stratified according to baseline status, using the median value for each biomarker as the cut‐point. For all analyses, *p* < 0.05 was considered statistically significant.

## Results

### Study population and general characteristics

On the basis of B‐vitamin analysis performed on completion of the trial, we retrospectively identified that a small number of participants who had been randomized to treatment had biomarker values indicative of supplement use and had thus underreported B‐vitamin supplement usage at time of recruitment (*n* = 35). Therefore, these participants were subsequently excluded from all analyses on the basis that they did not meet the study criteria (Fig. 1). Of the valid participants randomized to intervention (*n* = 205; mean age 69.6 years; 40% male), 167 participants completed the intervention in full, representing an 81% completion rate (Fig. 1). Some 52% of the cohort had hypertension and 12% reported a diagnosis of diabetes (Table 1). Generally, the baseline characteristics of the treatment groups were similar, with no significant differences in weight, height, BMI, or waist‐to‐hip ratio. In addition, there were no significant differences in dietary intakes between the treatment groups at baseline (Table 1).

**Table 1:** Characteristics of OptiAge Trial Participants at Baseline (*n* = 205^a^)

	Active placebo (*n* = 102)	B‐vitamin treatment (*n* = 103)	*p* Value^b^,^c^
General characteristics			
Age (years)	70.6 (69.1, 72.0)	68.7 (67.3, 70.1)	0.085
Sex, *n* (% male)	42 (41)	39 (38)	0.732
BMI (kg/m^2^)	28.6 (27.6, 29.6)	28.2 (27.3, 29.1)	0.619
Waist/hip ratio (cm)	0.91 (0.90, 0.93)	0.91 (0.90, 0.93)	0.874
Timed up‐and‐go (seconds)	9.0 (8.6, 9.3)	9.4 (8.5, 10.2)	0.585
Health and lifestyle			
Alcohol (units/week)^d^	4.2 (3.0, 5.5)	4.6 (3.5, 5.7)	0.594
Smoking status			
Current, *n* (%)	1 (1)	4 (4)	0.368
Past, *n* (%)	50 (49)	46 (45)	
Never, *n* (%)	51 (50)	52 (51)	
Supplement users, *n* (%)	45 (45)	39 (38)	0.440
Physical self‐maintenance score	22.6 (21.9, 23.4)	23.3 (23.0, 23.6)	0.050
Instrumental activities of daily living	27.5 (27.2, 27.8)	27.3 (26.9, 27.6)	0.053
Fallen within 12 months, *n* (%)	23 (23)	21 (20)	0.836
PASE score	118 (106, 129)	117 (105, 130)	0.537
General medical			
Systolic BP (mm Hg)	135 (131, 138)	134 (130, 137)	0.815
Diastolic BP (mm Hg)	78 (76, 80)	78 (76, 80)	0.793
Hypertension,^e^ *n* (%)	33 (33)	44 (43)	0.164
Previous heart attack, *n* (%)	4 (4)	5 (5)	0.473
Diabetes, *n* (%)	12 (12)	12 (12)	0.608
Dietary intake^f^			
Fortified food consumer,^g^ *n* (%)	29 (35)	37 (39)	0.609
Energy (MJ/d)	7.29 (6.91, 7.68)	7.39 (7.02, 7.76)	0.628
Vitamin D (μg/d)	3.2 (2.7, 3.7)	2.7 (2.4, 3.1)	0.379
DFE^h^ (μg/d)	210 (194, 226)	219 (205, 234)	0.378
Vitamin B12 (μg/d)	5.2 (4.6, 5.8)	4.8 (4.3, 5.3)	0.479
Vitamin B6 (mg/d)	1.7 (1.5, 1.8)	1.6 (1.5, 1.8)	0.931
Riboflavin (mg/d)	1.5 (1.4, 1.6)	1.5 (1.4, 1.6)	0.819

1 2

^a^ UU (*n* = 109, mean age 74.3 years, 46% male); UCD (*n* = 96, mean age 64.4 years, 32% male).

^b^ Differences between groups were assessed using an independent samples *t* test or chi‐square analysis (categorical variables); *p* < 0.05.

^c^ Differences between groups for Health and Lifestyle and General medical characteristics were assessed using ANCOVA (adjusted for age); *p* < 0.05.

^d^ Alcohol (units/week); 1 unit equates with 25 mL spirits, 220 mL beer, or 85 mL wine.

^e^ Hypertension defined as systolic BP of ≥140 and/or a diastolic BP of ≥90 mm Hg.

^f^ Dietary data collected via a 4‐day food diary was available for 87% of the cohort (*n* = 84 active placebo; *n* = 94 B‐vitamin treatment).

^g^ Participants who consumed foods fortified with B‐vitamins at least once per week.

^h^ Dietary folate equivalents were calculated as (natural folate [μg]) + (1.7 × synthetic folic acid [μg]).

### Bone health at baseline

There were no differences in BMD at any of the three sites between the treatment groups (Table 2), and the prevalence of osteopenia/osteoporosis was similar (61% versus 64%, respectively). Regression analysis showed that sex and BMI were significant determinants of BMD at all three measured sites (Table 3), although BMI was associated with osteopenia/osteoporosis (Table 4). Surprisingly, regression analysis also showed that higher alcohol consumption was associated with higher BMD (Table 3) and a reduced risk of osteopenia/osteoporosis (Table 4).

**Table 2:** Bone Health Characteristics and Bone Mineral Density at Baseline

	Active placebo (*n* = 102)	B‐vitamin treatment (*n* = 103)	*p* Value^a^,^b^
Bone health characteristics			
Previous fracture (any type), *n* (%)	45 (44)	45 (44)	1.000
Parental hip fracture, *n* (%)	17 (17)	17 (17)	0.601
Glucocorticoid use >3 months, *n* (%)	6 (6)	3 (3)	0.495
Bisphosphonate use, *n* (%)	6 (6)	9 (9)	0.592
Vitamin D status			
Deficient (<50 nmol/L), *n* (%)	46 (45)	41 (40)	
Bone mineral density (g/cm^2^)			
Total hip	0.970 (0.941, 1.000)	0.968 (0.940, 0.996)	0.353
*T*‐score	−0.5 (−0.8, −0.3)	−0.5 (−0.7, −0.3)	
Femoral neck	0.891 (0.866, 0.917)	0.903 (0.878, 0.928)	0.938
*T*‐score	−1.0 (−1.2, −0.8)	−0.9 (−1.1, −0.7)	
Lumbar spine	1.120 (1.084, 1.156)	1.132 (1.093, 1.171)	0.170
*T*‐score	−0.6 (−0.9, −0.3)	−0.5 (−0.8, −0.2)	
Osteoporotic,^c^ *n* (%)	16 (16)	14 (14)	0.821
Osteopenic/osteoporotic,^d^ *n* (%)	61 (60)	66 (64)	0.627

Data presented are mean (95% confidence interval [CI]) unless otherwise indicated.

^a^ Differences between groups were assessed using an independent samples *t* test (continuous variables) or chi‐square analysis (categorical variables); *p* < 0.05.

^b^ Differences between groups for bone mineral density were assessed by using ANCOVA (adjusted for age); *p* < 0.05.

^c^ Overall classification of osteoporosis is based on the lowest *T*‐score at any of the three measured sites: normal bone health *T*‐score ≥ −1.0; osteopenia *T*‐score < −1.0 to > −2.5; osteoporosis *T*‐score ≤ −2.5.^(^ ^23^ ^)^

^d^ Osteopenia and osteoporosis combined.

**Table 3:** Determinants of Bone Mineral Density in Older Adults at Baseline

Standardized beta (β) values are presented; *n* = 205	Bone mineral density (g/cm^2^)					
	Total hip		Femoral neck		Lumbar spine	
	β	*p* ^a^	β	*p* ^a^	β	*p* ^a^
Age (years)	−0.066	0.549	−0.083	0.490	0.063	0.553
Female sex	−0.370	**<0.001**	−0.285	**0.001**	−0.281	**<0.001**
BMI (kg/m^2^)^b^	0.340	**<0.001**	0.259	**0.003**	0.281	**<0.001**
Recruitment center^c^	−0.101	0.327	−0.160	0.160	0.015	0.884
Timed up‐and‐go (seconds)	0.054	0.546	0.025	0.799	0.055	0.529
Serum total homocysteine (μmol/L)	−0.050	0.530	−0.042	0.628	0.055	0.474
Serum 25(OH)D (nmol/L)	0.043	0.566	0.017	0.833	−0.024	0.745
Alcohol (units/week)^d^	0.261	**0.002**	0.219	**0.018**	0.374	**<0.001**
Smoking (past/current)	0.016	0.833	0.015	0.857	−0.031	0.682
Previous bone fracture	−0.086	0.239	−0.069	0.388	−0.053	0.456

BMI = body mass index; 25(OH)D = 25‐hydroxyvitamin D.

^a^ Data analyzed by multiple linear regression (with mutual adjustment for all other variables in the model) on log‐transformed data where appropriate; *p* < 0.05.

^b^ When the components of BMI were entered in the model separately as an alternative approach, weight remained highly significant as a determinant of BMD at all sites, whereas height was generally not significantly associated with BMD and other variables remained unchanged.

^c^ Recruitment center refers to either Ulster University or University College Dublin.

^d^ Alcohol (units/week); 1 unit equates with 25 mL spirits, 220 mL beer or 85 mL wine.

**Table 4:** Factors Associated With Risk of Osteopenia/Osteoporosis in Older Adults at Baseline

Standardized beta (β) values are presented; *n* = 205	β	OR	95% CI	*p* Value^a^
Age (years)	0.025	1.03	0.96–1.09	0.453
Female sex	0.540	1.72	0.88–3.34	0.112
BMI (kg/m^2^)	−0.162	0.85	0.79–0.92	**<0.001**
Recruitment center^b^	0.146	1.16	0.47–2.86	0.752
Timed up‐and‐go (seconds)	0.033	1.03	0.87–1.22	0.705
Serum total homocysteine (μmol/L)	0.018	1.02	0.94–1.10	0.635
Serum 25(OH)D (nmol/L)	0.004	1.00	0.99–1.02	0.512
Alcohol consumer	−1.342	0.26	0.11–0.62	**0.002**
Smoking (past/current)	−0.029	0.97	0.5–1.89	0.931
Previous bone fracture	−0.038	0.96	0.5–1.87	0.911

BMI = body mass index; 25(OH)D = 25‐hydroxyvitamin D.

^a^ Data analyzed by binary logistic regression (with mutual adjustment for all other variables in the model) on log‐transformed data where appropriate; *p* < 0.05.

^b^ Comparing recruitment site Ulster University with University College Dublin (reference category).

### B‐vitamin biomarker status at baseline

At baseline, mean values for B‐vitamin biomarkers generally compared favorably with normal ranges with the exception of MMA, the mean value for which was in the range indicative of deficient B12 status (Table 5; pre‐intervention values). Although mean values for B‐vitamin biomarkers (apart from MMA) were within normal ranges, 17% of participants had low or deficient status of vitamin B12 (as determined by cB12 ≤ −0.5), 16% were deficient in serum folate (<10 nmol/L), 22% were deficient in vitamin B6 (plasma PLP < 30 nmol/L), and 29% had riboflavin status within the deficient range (EGRac; >1.40); data not shown. There were no significant associations at baseline between individual B‐vitamin biomarkers (or serum tHcy) and BMD at any site, in the total cohort or in males or females separately (data not shown).

**Table 5:** Response of Vitamin Biomarkers to Intervention With Low‐dose B‐Vitamins^a^

	Active placebo (*n* = 102)	B‐vitamin treatment (*n* = 103)	*p* Value^b^,^c^
Age (years)	70.6 (69.1, 72.0)	68.7 (67.3, 70.1)	0.092
Sex, *n* (% male)	41 (41)	39 (38)	0.798
Biomarkers			
Serum total vitamin B12 (pmol/L)			
Pre^d^	262 (244, 279)	246 (230, 262)	
Post	254 (237, 272)	343 (319, 367)	<0.001
Change	−7 (−18, 3)	97 (78, 115)	
Serum MMA (μmol/L)			
Pre^d^	0.28 (0.24, 0.31)	0.30 (0.24, 0.36)	
Post	0.30 (0.26, 0.34)	0.26 (0.20, 0.31)	<0.001
Change	0.02 (0.00, 0.05)	−0.05 (−0.07, −0.02)	
Serum folate (nmol/L)			
Pre^d^	16.1 (14.9, 17.2)	17.0 (15.7, 18.3)	
Post	22.2 (16.9, 27.5)	31.7 (23.5, 39.8)	<0.001
Change	6.1 (0.7, 11.5)	14.7 (6.5, 22.9)	
Plasma PLP (vitamin B6; nmol/L)			
Pre^d^	45.3 (41.5, 49.1)	44.6 (41.3, 47.8)	
Post	52.8 (44.8, 60.8)	213.7 (187.8, 239.5)	<0.001
Change	7.5 (0.3, 14.8)	169.1 (144.3, 193.9)	
Riboflavin (EGRac)			
Pre^d^	1.34 (1.31, 1.37)	1.36 (1.33, 1.39)	
Post	1.34 (1.30, 1.37)	1.18 (1.15, 1.21)	<0.001
Change	0.00 (−0.02, 0.02)	−0.18 (−0.21, −0.15)	
Serum total homocysteine (μmol/L)			
Pre^d^	13.8 (12.6, 15.0)	13.7 (12.8, 14.6)	
Post	13.7 (12.5, 14.9)	11.1 (10.3, 11.9)	<0.001
Change	−0.1 (−0.5, 0.3)	−2.5 (−3.1, −2.0)	
Serum 25(OH)D (nmol/L)			
Pre^d^	55.2 (49.4, 61.0)	57.5 (52.2, 62.7)	
Post	78.2 (72.5, 83.9)	83.4 (77.9, 89.0)	0.918
Change	23.0 (17.3, 28.8)	26.3 (20.4, 31.6)	

Data presented as mean (95% confidence interval [CI]) unless otherwise indicated.

EGRac = erythrocyte glutathione reductase activation coefficient; MMA = methylmalonic acid; PLP = pyridoxal‐5‐phosphate; 25(OH)D = 25‐hydroxyvitamin D.

^a^ The treatment capsule contained a combination of 10 μg vitamin D, along with B‐vitamins (200 μg folic acid, 10 μg vitamin B12, 10 mg vitamin B6, and 5 mg riboflavin). The “active” placebo capsule contained 10 μg vitamin D only. Both treatments were administered to participants daily.

^b^ Differences in general characteristics between groups were assessed using an independent samples *t* test for continuous variables (age) and chi‐square analysis for categorical variables (sex); *p* < 0.05.

^c^ Biomarker responses to intervention were assessed by repeated measures ANOVA on an intention‐to‐treat basis. The *p* values shown for all biomarker results refer to time × treatment interaction; *p* < 0.05.

^d^ Cut‐points to indicate normal ranges for each of the biomarkers were as follows: serum total B12 > 221 pmol/L^(^ ^13^ ^)^; serum MMA <0.27 μmol/L^(^ ^8^ ^)^; serum folate >10 nmol/L^(^ ^35^ ^)^; PLP > 30 nmol/L^(^ ^32^ ^)^; EGRac <1.40^(^ ^33^ ^)^; serum total homocysteine <16 μmol/L^(^ ^36^ ^)^; and serum 25‐hydroxyvitamin D > 50 nmol/L.^(^ ^37^ ^)^

### Verification of target doses and compliance with the study protocol

On analysis of the treatment capsules at baseline, the vitamin doses were found to vary marginally from the target doses: folic acid 200 μg (184 μg); vitamin B12 10 μg (9.6 μg); vitamin B6 10 mg (10.2 mg); riboflavin 5 mg (4.3 mg); and vitamin D 10 μg (11.2 μg). Analysis of the “active” placebo capsule showed values similar to the targeted vitamin dose: vitamin D 10 μg (10.8 μg) and negligible amounts of B‐vitamins. Overall, there was generally excellent participant compliance with the intervention protocol (estimated by pill counting to be 88%).

### Effects of intervention on B‐vitamin biomarkers and change in BMD

B‐vitamin intervention over the 2‐year period resulted in significant increases in serum folate, serum vitamin B12, and plasma PLP, and corresponding decreases in EGRac, serum MMA, and serum tHcy concentrations (Table 5).

**Fig. 2:**
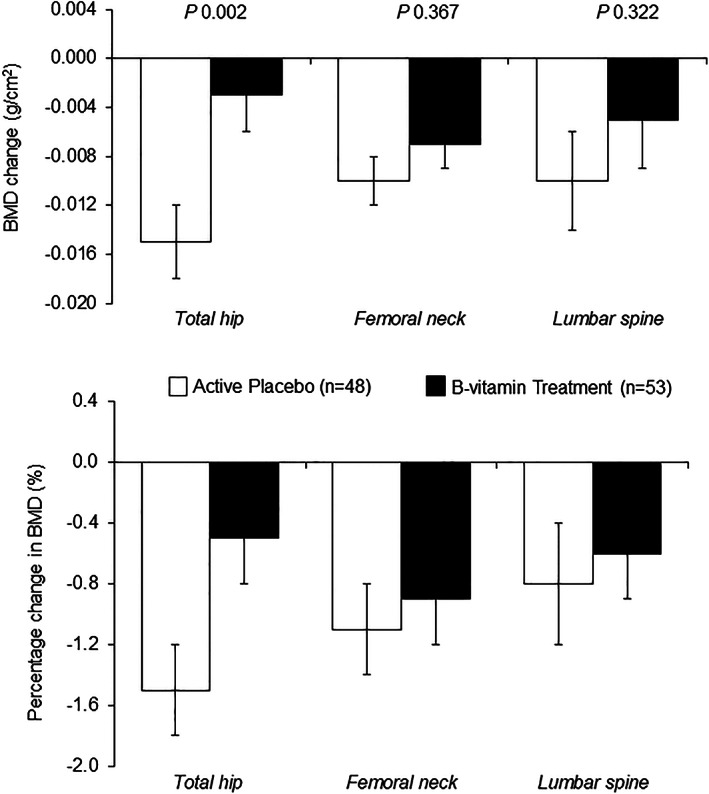
Change (upper plot^a^) and percentage change (lower plot^b^) in bone mineral density (BMD) after 2 years of supplementation with low‐dose B‐vitamins^c^ in participants with low vitamin B12 status at baseline (as defined by serum total B12 < median value of 246 pmol/L). ^a^Values are adjusted mean ± SEM. Change was calculated as the BMD value post‐intervention minus pre‐intervention. Differences between groups were assessed using ANCOVA (adjusted for age and sex); *p* < 0.05. ^b^Values are mean ± SEM. Percentage change (%) was calculated as the BMD value post‐intervention minus pre‐intervention expressed as a percentage relative to the pre‐intervention BMD value. ^c^The treatment capsule contained a combination of 10 μg vitamin D, along with B‐vitamins (200 μg folic acid, 10 μg vitamin B12, 10 mg vitamin B6, and 5 mg riboflavin). The “active” placebo capsule contained 10 μg vitamin D only. Both treatments were administered to participants daily. SEM = standard error of mean.

**Fig. 3:**
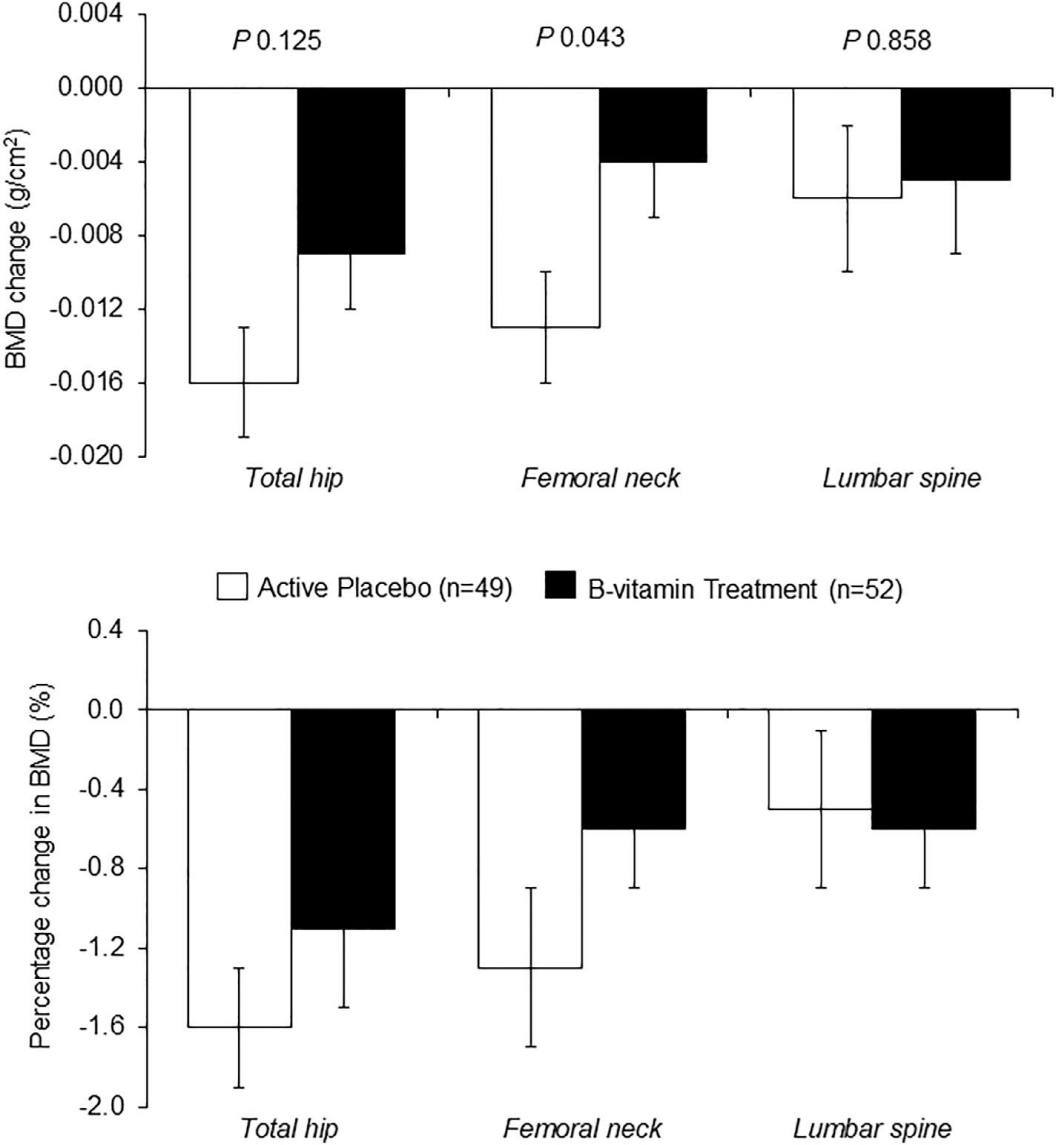
Change (upper plot^a^) and percentage change (lower plot^b^) in bone mineral density (BMD) after 2 years of supplementation with low‐dose B‐vitamins^c^ in participants with low vitamin B12 status at baseline (as defined by serum methylmalonic acid [MMA] ≥ median value of 0.22 μmol/L). ^a^Values are adjusted mean ± SEM. Change was calculated as the BMD value post‐intervention minus pre‐intervention. Differences between groups were assessed using ANCOVA (adjusted for age and sex); *p* < 0.05. ^b^Values are mean ± SEM. Percentage change (%) was calculated as the BMD value post‐intervention minus pre‐intervention expressed as a percentage relative to the pre‐intervention BMD value. ^c^The treatment capsule contained a combination of 10 μg vitamin D, along with B‐vitamins (200 μg folic acid, 10 μg vitamin B12, 10 mg vitamin B6, and 5 mg riboflavin). The “active” placebo capsule contained 10 μg vitamin D only. Both treatments were administered to participants daily. SEM = standard error of mean.

Regardless of treatment allocation, BMD at all three sites decreased by approximately 1% (ranging from −0.7% to −1.4%) over the 2‐year intervention (Table 6). Overall, there was no significant effect of intervention with B‐vitamins on BMD at any site, either in the cohort as a whole or when examined in males and females separately, albeit BMD was lower at both time points at all sites in females (Supplemental Table S1). However, when the 2‐year change in BMD was examined among participants with lower baseline vitamin B12 status (<median serum B12 of 246 pmol/L or ≥median MMA of 0.22 μmol/L), B‐vitamin intervention compared with placebo significantly decreased the rate of BMD decline at the total hip (Fig. 2) and at the femoral neck (Fig. 3) after adjustments for age and sex.

**Table 6:** Responses of BMD to Intervention With Low‐Dose B‐Vitamins for 2 Years^a^

	Active placebo (*n* = 102)	B‐vitamin treatment (*n* = 103)	*p* Value^b^	*p* Value^c^
Age (years)	70.6 (69.1, 72.0)	68.7 (67.3, 70.1)	0.085	
Sex, *n* (% male)	42 (41)	39 (38)	0.732	
BMI (kg/m^2^)	28.6 (27.6, 29.6)	28.2 (27.3, 29.1)	0.619	
Alcohol (units/week)^d^	4.2 (3.0, 5.5)	4.6 (3.5, 5.7)	0.303	
Bone mineral density (g/cm^2^)				
Total hip				
Pre	0.970 (0.941, 1.000)	0.968 (0.940, 0.996)		
Post	0.962 (0.933, 0.991)	0.957 (0.928, 0.985)		
Change	−0.009 (−0.013, −0.004)	−0.012 (−0.016, −0.007)	0.353	0.391
% Change	−0.8 (−1.3, −0.4)	−1.2 (−1.7, −0.7)		
Femoral neck				
Pre	0.891 (0.866, 0.917)	0.903 (0.878, 0.928)		
Post	0.884 (0.859, 0.909)	0.895 (0.870, 0.921)		
Change	−0.008 (−0.013, −0.003)	−0.007 (−0.012, −0.002)	0.938	0.788
% Change	−0.9 (−1.3, −0.3)	−0.8 (−1.4, −0.2)		
Lumbar spine				
Pre	1.120 (1.084, 1.156)	1.132 (1.093, 1.171)		
Post	1.111 (1.076, 1.146)	1.117 (1.157, 1.110)		
Change	−0.010 (−0.017, −0.003)	−0.014 (−0.020, −0.007)	0.170	0.460
% Change	−0.7 (−1.2, −0.1)	−1.4 (−2.1, −0.7)		

Data presented as mean (95% confidence interval [CI]), apart from Change, which is presented as adjusted mean (95% CI).

BMI = body mass index; BMD = bone mineral density.

^a^ The treatment capsule contained a combination of 10 μg vitamin D, along with B‐vitamins (200 μg folic acid, 10 μg vitamin B12, 10 mg vitamin B6, and 5 mg riboflavin). The “active” placebo capsule contained 10 μg vitamin D only. Both treatments were administered to participants daily.

^b^ Differences between groups were assessed using an independent samples *t* test for continuous variables and chi‐square analysis for categorical variables (sex); *p* < 0.05.

^c^ Change in BMD was assessed on an intention‐to‐treat basis by ANCOVA (adjusted for age, sex, BMI, alcohol intake units/week), *p* < 0.05; analysis was conducted on log‐transformed data where appropriate.

^d^ Alcohol (units/week); 1 unit equates with 25 mL spirits, 220 mL beer, or 85 mL wine.

No other significant effects of intervention on BMD were observed when participants with lower status of any of the other B‐vitamins or in those with elevated tHcy were analyzed. This was found to be the case when the data were examined using the median biomarker value as a cut point to identify those with lower status (serum folate, <16.3 nmol/L; plasma PLP, <43.5 nmol/L; EGRac, ≥1.33; tHcy, ≥12.7 μmol/L). Likewise, no effects of intervention on BMD were observed when established deficiency cut‐offs for each biomarker were used: serum folate (<10 nmol/L),^(^ ^35^ ^)^ PLP (<30 nmol/L),^(^ ^32^ ^)^ EGRac (>1.40),^(^ ^33^ ^)^ tHcy (>16 μmol/L),^(^ ^36^ ^)^ serum 25(OH)D (<50 nmol/L)^(^ ^37^ ^)^; data not shown.

## Discussion

This study investigated the effect of intervention with low‐dose B‐vitamins on BMD in adults aged ≥50 years in an RCT using an active placebo, whereby both treatment groups received vitamin D. The results showed that daily supplementation for 2 years with combined folic acid, vitamin B12, vitamin B6, and riboflavin had no overall effect on BMD. Subgroup analyses, however, showed that B‐vitamin intervention resulted in reducing the rate of BMD decline among those with lower vitamin B12 status at baseline (as indicated by either serum B12 or MMA concentrations).

As expected, after 2 years of intervention, all B‐vitamin biomarkers improved and tHcy concentrations decreased. However, there was no significant effect of intervention on BMD at any measured site. Likewise, one small trial in osteoporotic patients (*n* = 47) investigating B‐vitamin intervention at much higher doses (folic acid, 2.5 mg; vitamin B12, 500 μg; vitamin B6, 25 mg) also reported no effect on BMD.^(^ ^38^ ^)^ Of greater note, the large VITATOPS trial in patients with recent stroke (*n* = 8164) also found no effect of treatment with similarly high doses of folic acid, B6, and B12 on osteoporotic fracture incidence after 2.8 years.^(^ ^39^ ^)^ Similarly, the B‐PROOF trial in Dutch older adults found no effect on osteoporotic fracture incidence of combined vitamin B12 (500 μg) and folic acid (400 μg) supplementation for 2 years, and like the current study, vitamin D was included in both treatment arms.^(^ ^10^ ^)^ Furthermore, a meta‐analysis of RCTs found no effect of B‐vitamin intervention on fracture risk in adults.^(^ ^40^ ^)^ Thus, the limited randomized trials to date show no benefits of B‐vitamins on fracture incidence or BMD, albeit it should be noted that most trials were not designed to examine bone health as a primary outcome or in the general population.

Despite no overall effect of B‐vitamin intervention on BMD in the present study, our subgroup analysis found beneficial effects on BMD in those with lower baseline vitamin B12 status. In participants with serum B12 below the median value of 246 pmol/L or with MMA above the median value of 0.22 μmol/L at baseline, B‐vitamin intervention decreased the 2‐year BMD decline at the total hip and at the femoral neck. No similar effect of intervention on BMD was shown among participants with lower baseline status of folate, vitamin B6, or riboflavin or in those with higher tHcy concentrations, suggesting an independent role for vitamin B12 in bone health. In broad agreement with a B12‐specific effect on bone health, lower serum B12 concentrations (<207 pmol/L; lowest quintile) were associated with accelerated bone loss at the hip in women ≥65 years in a large prospective study in the United States.^(^ ^41^ ^)^ Likewise, B12 concentrations defined as deficiency (<148 pmol/L) were associated with lower BMD at the hip in males in the Framingham Osteoporosis Study (*n* = 2576).^(^ ^11^ ^)^ Furthermore, in nationally representative samples from the United States, increasing concentrations of MMA (a functional biomarker of B12) were associated with lower BMD and higher osteoporosis risk at the hip in older adults^(^ ^42^ ^)^ and at the lumbar spine in women ≥50 years.^(^ ^43^ ^)^ Given that MMA is considered a specific and sensitive indicator of tissue vitamin B12 status,^(^ ^8^ ^)^ the current and previous findings support a specific effect of vitamin B12 on bone health. In contrast, low plasma folate (but not B12) was associated with increased fracture risk in Norwegian adults of >60 years.^(^ ^16^ ^)^ The inconsistencies between studies in observed associations of vitamin B12 with bone outcomes could be attributable to the use of different biomarkers and/or different cut‐off values to define B12 deficiency. Given the limitations of using a sole B12 biomarker, experts now recommend the use (or combination) of two or more biomarkers to assess B12 status,^(^ ^8^, ^31^ ^)^ but few bone studies provide this. In addition, vitamin B12 status may differ between populations, both geographically and socioeconomically, reflecting differences in intakes of animal foods, the only source of B12 for humans,^(^ ^8^ ^)^ which may also influence the relationship of B12 with bone health outcomes. Nonetheless, the current results suggest that any beneficial effect of B‐vitamin intervention will be most evident in those with lower vitamin B12 status.

A decrease in BMD with age is well documented,^(^ ^44^ ^)^ with osteoporosis developing when negative bone balance exists, distinguishable by an imbalance in the coupling of bone formation and resorption by osteoblasts and osteoclasts, respectively.^(^ ^45^ ^)^ Regardless of treatment allocation, BMD at all three sites declined by approximately 1% (ranging from −0.7% to −1.4%) over the 2‐year intervention. Our observations are comparable with rates of BMD decline observed in other studies.^(^ ^46^, ^47^ ^)^ Through the use of an active placebo, with vitamin D administered to both treatment arms, all participants likely benefited from the current intervention given that vitamin D, combined with calcium, has a well‐documented role in bone health,^(^ ^5^, ^6^, ^7^ ^)^ with evidence that higher serum 25(OH)D is associated with greater BMD in older adults.^(^ ^48^ ^)^ Given that both groups received vitamin D, it can be assumed that any effect of B‐vitamin intervention on BMD decline was independent of vitamin D.

The mechanism to explain the role of B‐vitamins in bone health as suggested here and elsewhere is unclear. The current findings, however, point to an independent role of vitamin B12 in bone health, as observed in previous in vitro and in vivo studies. One small trial found lower concentrations of bone formation markers (alkaline phosphatase and osteocalcin) in vitamin B12‐deficient patients compared with controls and a subsequent rise in skeletal alkaline phosphatase after B12 supplementation—an effect found only in the B12‐deficient patients.^(^ ^49^ ^)^ Additional in vitro evidence also showed a stimulatory effect of vitamin B12 on alkaline phosphatase,^(^ ^50^ ^)^ suggesting B12 may have a protective effect on the balance between bone formation and resorption. Further mechanistic studies are required to elucidate the effects of vitamin B12, and indeed related B‐vitamins, on bone health.

The current findings will have greatest relevance for aging adults who are at increased risk of vitamin B12 deficiency, despite typically having dietary B12 intakes, which exceed the current recommendation of 4 μg/d, as set by the European Food Safety Authority.^(^ ^22^, ^51^ ^)^ Using a comprehensive suite of B12 biomarkers, we recently reported a high prevalence of B12 deficiency in older Irish adults, particularly among those identified with atrophic gastritis or taking PPI medications, among whom 38% and 21%, respectively, were B12 deficient.^(^ ^14^ ^)^ Notably, the intakes of crystalline vitamin B12 (10 μg/d), shown in the present study to be effective in optimizing B12 status and reducing the rate of BMD decline, are achievable through intervention with B12‐fortified foods.^(^ ^52^ ^)^ However, fortified foods currently available in Ireland and the United Kingdom are typically low in added vitamin B12.^(^ ^53^ ^)^ Thus, consideration should be given to encouraging the food industry to increase the B12 content of fortified products in order to help optimize B12 status, with consequent potential benefits for bone and other health outcomes associated with deficient status.

The study is not without its limitations. Although at screening we excluded those reporting B‐vitamin supplement usage and high intakes of fortified foods, participants were not initially selected using biomarker evidence of low baseline B‐vitamin status. Instead, we performed subgroup analysis of the results to investigate the effect of intervention in this group, an approach that resulted in smaller sample sizes with reduced statistical power. Targeted recruitment to this trial of adults with biomarker evidence of low/deficient status may have enabled the detection of more significant effects of B‐vitamin intervention on BMD, albeit this approach may raise ethical concerns in relation to delaying treatment for the 2‐year duration of the trial in those identified with deficient biomarker values.

This study also has a number of strengths. The randomized trial design enabled a cause‐and‐effect relationship between vitamin B12 and BMD to be shown in those with lower B12 status. Furthermore, we performed our trial in a general adult population and measured BMD as the primary outcome, whereas the majority of previous trials were in patient groups and were not designed to measure bone health as a primary outcome.^(^ ^54^, ^55^, ^56^, ^57^ ^)^ In addition, we used robust endpoints with BMD assessment using DXA scanning, widely considered as the gold standard for monitoring changes in BMD over time.^(^ ^58^ ^)^ Also, this is the first human trial to investigate the effect of all relevant B‐vitamins, including riboflavin, which is rarely assessed in human studies,^(^ ^59^ ^)^ thus enabling a more complete investigation of the potential impact of one‐carbon metabolism on bone health. A particular strength was the use of three biomarkers of B12 to assess status, given the inherent limitations of individual B12 biomarkers when used alone.^(^ ^30^, ^31^ ^)^ Finally, the current trial used doses of B‐vitamins that potentially are achievable through dietary means via a combination of natural and fortified food sources, whereas previous trials have typically used micronutrient doses up to 100‐fold higher than current dietary recommendations and, therefore, not achievable through dietary means, thus greatly limiting the translation of their findings into nutrition recommendations and policy for better bone health and prevention of osteoporosis.

In conclusion, the findings indicate that although B‐vitamin intervention for 2 years had no overall effect on BMD, intervention at levels within dietary ranges to improve B‐vitamin status may have specific benefits for bone health in adults with lower vitamin B12 status. These findings require further investigation in a randomized trial targeting participants at greatest risk of low or deficient B12 status, including those with atrophic gastritis or using PPI medications. Given the high prevalence of vitamin B12 deficiency among older populations, the current results, if confirmed, could have important implications for bone health and prevention of osteoporosis.

## Disclosures

All authors state that they have no conflicts of interest.

## AUTHOR CONTRIBUTIONS

**Michelle Clements:** Formal analysis; investigation; methodology; project administration; writing – original draft. **Maria Heffernan:** Investigation; methodology; project administration; writing – review and editing. **Mary Ward:** Conceptualization; supervision; writing – original draft. **Leane Hoey:** Writing – original draft. **Leanne C Doherty:** Investigation; methodology; project administration; writing – review and editing. **Roberta Hack Mendes:** Investigation; methodology; project administration; writing – review and editing. **Michelle M Clarke:** Conceptualization; writing – review and editing. **Catherine F Hughes:** Writing – original draft. **Ingrid Love:** Investigation. **Shauna Murphy:** Investigation. **Eilish McDermott:** Investigation. **Jennifer Grehan:** Investigation. **Adrian McCann:** Investigation; writing – review and editing. **Liadhan B McAnena:** Investigation; writing – review and editing. **JJ Strain:** Conceptualization; writing – review and editing. **Lorraine Brennan:** Conceptualization; funding acquisition; supervision; writing – review and editing. **Helene McNulty:** Conceptualization; funding acquisition; supervision; writing – original draft.

## Supporting information


**Supplemental Table S1.** Responses of BMD to Intervention With Low‐Dose B‐Vitamins for 2 Years in Males and Females

## Data Availability

Data described in the manuscript, code book, and analytic code will be made available upon request, pending application and approval.

## Footnotes

1 ANCOVA = analysis of covariance; BP = blood pressure; BMI = body mass index; DFE = dietary folate equivalents; PASE = physical activity scale for the elderly; UU = Ulster University; UCD = University College Dublin.

2 Data presented are mean (95% confidence interval [CI]) unless otherwise indicated.
