# Supplementary material for: A 2‐Year Randomized Controlled Trial With Low‐Dose B‐Vitamin Supplementation Shows Benefits on Bone Mineral Density in Adults With Lower B12 Status
Source: J Bone Miner Res. 2022 Oct 14;37(12):2443–55. doi: 10.1002/jbmr.4709 (PMC10092614; doi:10.1002/jbmr.4709)
Supplement: Supplementary file 1 — Supplemental Table S1. Responses of BMD to Intervention With Low‐Dose B‐Vitamins for 2 Years in Males and Females [file JBMR-37-2443-s001.pdf]

Supplemental Table 1 Responses of BMD to intervention with low-dose B-vitamins for 2 years in males and females[1]

	**Males (n=81)**			**Females (n=124)**		
	**Active Placebo (n=42)**	**B-vitamin Treatment (n=39)**	***P* value[2,3]**	**Active Placebo (n=60)**	**B-vitamin Treatment (n=64)**	***P* value[2,3]**
Age (years)	72.0 (69.4, 74.6)	70.2 (68.1, 72.2)	0.331	69.5 (67.8, 71.3)	67.8 (65.9, 69.8)	0.180
BMI (kg/m^2)	29.3 (27.8, 30.8)	28.1 (26.9, 29.4)	0.259	28.1 (26.8, 29.5)	28.3 (27.1, 29.5)	0.178
Baseline B-vitamin biomarkers						
Serum folate (nmol/L)	15.6 (13.5, 17.8)	16.6 (14.7, 18.5)	0.322	16.4 (15.1, 17.7)	17.2 (15.4, 19.0)	0.823
Serum total vitamin B12 (pmol/L)	235 (211, 259)	243 (219, 268)	0.703	280 (256, 304)	247 (226, 268)	0.039
Serum MMA (µmol/L)	0.31 (0.25, 0.37)	0.34 (0.21, 0.47)	0.758	0.25 (0.21, 0.29)	0.28 (0.23, 0.33)	0.425
Plasma PLP (vitamin B6, nmol/L)	42.3 (36.2, 49.0)	47.4 (41.7, 53.1)	0.125	47.2 (42.5, 51.9)	42.9 (38.9, 46.8)	0.208
Riboflavin (EGRac)	1.33 (1.27, 1.40)	1.36 (1.31, 1.41)	0.434	1.34 (1.31, 1.37)	1.36 (1.31, 1.41)	0.608
Serum total homocysteine (µmol/L)	15.5 (12.9, 18.1)	14.0 (12.4, 15.6)	0.457	12.6 (11.8, 13.5)	13.4 (12.3, 14.5)	0.418
Bone mineral density (g/cm^2)						
Total hip						
Pre	1.043 (0.996, 1.089)	1.043 (1.004, 1.082)		0.914 (0.881, 0.946)	0.913 (0.881, 0.945)	
Post	1.036 (0.990, 1.082)	1.037 (0.995, 1.079)		0.905 (0.873, 0.937)	0.899 (0.867, 0.930)	
Change	-0.007 (-0.014, 0.000)	-0.007 (-0.015, 0.001)	0.913	-0.008 (-0.015, -0.002)	-0.014 (-0.020, -0.008)	0.200
% change	*-0.6 (-1.2, 0.0)*	*-0.6 (-1.5, 0.3)*		*-0.9 (-1.6, -0.2)*	*-1.5 (-2.2, -0.9)*	
Femoral neck						
Pre	0.940 (0.896, 0.986)	0.957 (0.917, 0.996)		0.859 (0.831, 0.888)	0.872 (0.839, 0.904)	
Post	0.939 (0.895, 0.984)	0.957 (0.917, 0.997)		0.849 (0.820, 0.877)	0.860 (0.828, 0.892)	
Change	-0.003 (-0.010, 0.004)	0.001 (-0.007, 0.008)	0.507	-0.011 (-0.018, -0.003)	-0.012 (-0.019, -0.004)	0.937
% change	*-0.1 (-0.9, 0.7)*	*0.0 (-0.6, 0.6)*		*-1.2 (-2.0, -0.5)*	*-1.3 (-2.2, -0.3)*	
Lumbar spine						
Pre	1.247 (1.180, 1.314)	1.267 (1.195, 1.338)		1.059 (1.009, 1.109)	1.062 (1.017, 1.107)	
Post	1.240 (1.176, 1.305)	1.256 (1.180, 1.331)		1.053 (1.004, 1.101)	1.046 (1.000, 1.090)	
Change	-0.008 (-0.023, 0.006)	-0.009 (-0.024, 0.006)	0.940	-0.008 (-0.017, 0.001)	-0.015 (-0.023, -0.007)	0.219
% change	*-0.4 (-1.3, 0.5)*	*-1.0 (-2.4, 0.4)*		*-0.5 (-1.3, 0.2)*	*-1.5 (-2.3, -0.6)*	

Data presented as mean (95% CI), apart from Change which is presented as adjusted mean (95% CI).

On-line Supplementary Material

[1]The treatment capsule contained a combination of 10µg vitamin D, along with B-vitamins (200µg folic acid, 10µg vitamin B12, 10mg vitamin B6 and 5mg riboflavin). The 'active' placebo capsule contained 10µg vitamin D only. Both treatments were administered to participants daily.
[2]Differences between groups were assessed using an independent samples t-test for continuous variables; $P < 0.05$.
[3]Change in BMD was assessed on an intention-to-treat basis by ANCOVA (adjusted for age, sex, BMI, alcohol intake units/week), $P < 0.05$; Analysis was conducted on log-transformed data where appropriate.

Abbreviations: BMI, body mass index; BMD, bone mineral density; EGRac, erythrocyte glutathione reductase activation coefficient; MMA, methylmalonic acid; PLP, pyridoxal-5-phosphate
